# microRNA-148a dysregulation discriminates poor prognosis of hepatocellular carcinoma in association with USP4 overexpression

**DOI:** 10.18632/oncotarget.1920

**Published:** 2014-04-25

**Authors:** Mi Jeong Heo, Young Mi Kim, Ja Hyun Koo, Yoon Mee Yang, Jihyun An, Sook-Kyung Lee, Seung Jin Lee, Kang Mo Kim, Joong-Won Park, Sang Geon Kim

**Affiliations:** ^1^ College of Pharmacy and Research Institute of Pharmaceutical Sciences, Seoul National University, Seoul, Korea; ^2^ College of Pharmacy, Hanyang University, Ansan, Gyeonggido, Korea; ^3^ Department of Internal Medicine, Asan Liver Center, Asan Medical Center, University of Ulsan College of Medicine, Seoul, Korea; ^4^ Center for Liver Cancer, National Cancer Center, Goyang, Korea; ^5^ College of Pharmacy, Gachon University, Incheon, Korea

**Keywords:** hepatocellular carcinoma, USP4, S1P1, miR-148a, migration, growth

## Abstract

Hepatocellular carcinoma (HCC) is classified as a poor prognostic tumor, and becomes frequently aggressive. MicroRNAs emerge as key contributors to tumor progression. This study investigated whether miR-148a dysregulation differentiates poor prognosis of HCC, exploring new targets of miR-148a. miR-148a dysregulation discriminated not only the overall survival and recurrence free survival rates of HCC, but the microvascular invasion. In the human HCC samples, ubiquitin specific protease 4 (USP4) and sphingosine 1-phosphate receptor 1 (S1P1) were up-regulated as the new targets of miR-148a. USP4 and S1P1 were up-regulated in mesenchymal-type liver-tumor cells with miR-148a dysregulation, facilitating migration and proliferation of tumor cells. The inverse relationship between miR-148a and the identified targets was verified in a tumor xenograft model. In the analysis of human samples, the expression of USP4, but not S1P1, correlated with the decrease of miR-148a. In a heterotropic patient-derived HCC xenograft model, USP4 was also overexpressed in G1 and G2 tumors when miR-148a was dysregulated, reflecting the closer link between miR-148a and USP4 for a shift in the expansion phase of tumorgraft. In conclusion, miR-148a dysregulation affects the poor prognosis of HCC. Of the identified targets of miR-148a, USP4 overexpression may contribute to HCC progression towards more aggressive feature.

## INTRODUCTION

Hepatocellular carcinoma (HCC) belongs to common and aggressive human malignancies and is one of the leading causes of death by tumor worldwide. The high mortality rate of HCC is caused by frequent tumor metastasis, postsurgical recurrence, and late detection at advanced stages [1]. However, good diagnostic markers, drug targets and therapeutic strategies are still insufficient for successful treatment of HCC. MicroRNAs (miRNAs) modulate apoptosis, proliferation, migration and polarity of cells by changing target mRNA stability or translation [2]. In addition, miRNAs emerge as key contributors to tumor progression because of the ability to control multiple targets and alter biological functions [3]. Moreover, due to their stability, miRNAs are considered as useful tumor biomarkers [4]. miR-148a belongs to abundant miRNAs in hepatocytes, and is severely deregulated in several tumors including HCC [5,6]. However, whether dysregulation of miR-148a deteriorates the prognosis of HCC patients was elusive. Moreover, the molecules controlled by miR-148a in relation to HCC progression remained minimally known.

Ubiquitin specific proteases (USPs) regulate post-translational modification of proteins by inhibiting ubiquitination of its targets, affecting multiple biological processes such as cell cycle, DNA repair, and cell signaling pathways [7]. USP may be a key target for cancer therapy. Recently, it has been shown that several forms of USP (e.g., 4, 11 and 15) enhance transforming growth factor-β (TGF-β) signaling, which facilitates tumor cell invasion, migration, and aggressiveness [8,9]. However, USP expression in HCC and the upstream regulators leading to the changes in USP expression have been scarcely studied. Moreover, the contribution of USP to HCC progression has not been determined.

In tumor microenvironments, several lipid mediators promote tumor cell survival, inflammation and angiogenesis [10]. In particular, sphingosine 1-phosphate (S1P) may modulate tumor proliferation, protecting tumor cells from apoptosis by activating survival pathways presumably through S1P receptors (S1P1-5) [11]. In fact, S1P and S1P receptor levels are frequently increased in several tumors such as breast, prostate and Wilms tumors [12-14]. Nevertheless, the expression profile of S1P receptors and the role of specific S1P receptor type for HCC progression had not been explored.

This study investigated whether decrease of miR-148a affects the survival rates of patients with HCC, and the microvascular invasion (MVI). To elucidate the molecules responsible for HCC progression, we attempted to find miR-148a target(s) leading to the induction of metastatic phenotype. Using TargetScan algorithms, we extracted the ‘core genes’ having the greatest interaction partner genes with miR-148a and performed experiments using miR-148a mimic, antisense oligonucleotide (ASO), 3'-untranslated region (UTR) reporter, and constructs encoding for the targets. Here, we report the identification of USP4 and S1P1 as the direct targets of miR-148a. We also analyzed their expression levels in resected human samples. To determine the effect of USP4 or S1P1 overexpression on the progression of HCC in association with miR-148a dysregulation, we used a panel of tumor-derived cell lines and xenograft animal model. Recently, the patient-derived xenograft (PDX) model is considered as a useful tool for the study in cancer biology. PDX models closely represent the characteristics of original patient tumor because of their cell differentiation, morphology, and molecular signatures similarity as compared with cell line-derived xenografts [15,16]. Towards the end, we employed a PDX animal model to further assess the relationship between miR-148a and USP4 (or S1P1).

## RESULTS

### Poor prognosis of patients by decrease of miR-148a with the induction of USP4 and S1P1

**Figure 1:**
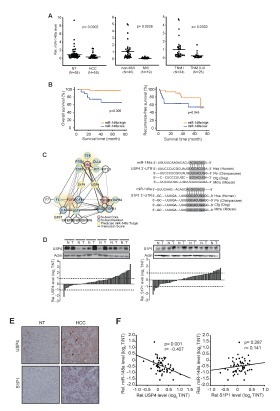
Survival and MVI rates of HCC patients in association with miR-148a dysregulation (A) qRT-PCR assays for miR-148a in HCC versus NT (left), HCC with MVI versus non-MVI (middle), or TNM I versus TNM II-III (right). The line indicates the mean. Statistical analysis was done by Student's t-test. (B) The overall survival rates and recurrence free survival rates of HCC patients with high or low miR-148a expression.(C) A gene interaction network. Filled colors indicate subset core (red) and member (blue), whereas border colors do predicted targets of miR-148a (yellow). Line thickness represents interaction text-mining score measured using STRING database (left). A sequence of miR-148a and its binding sites within the 3'-UTR regions of USP4 and S1P1 mRNAs were shown in the right.(D) Immunoblottings for USP4 and S1P1. Protein levels were measured in 59 pairs of primary HCCs (T) and their adjacent NTs (N). A log2 fold change more than 1 or less than −1 was considered overexpression or suppression. (E) Immunohistochemical analyses for USP4 or S1P1. Shown above are the representative figures (×200).(F) The correlation between miR-148a and USP4 or S1P1, as determined by Pearson analyses.

To understand the relationship between miR-148a expression and HCC progression, we first investigated miR-148a levels in HCC patients. The levels of miR-148a were diminished as compared to respective non-tumorous (NT) liver tissues (Figure 1A). More importantly, decrease of miR-148a further discriminated between HCC patients with MVI and those with non-MVI. Next, we divided HCC patients into two groups, low and high expression of miR-148a, according to the median level of miR-148a and analyzed survival rates of the HCC patients using Kaplan-Meier method. Interestingly, the overall survival rate was significantly lower in a group of HCC patients having low miR-148a expression (Figure 1B, left). Moreover, low miR-148a expression also significantly reduced the recurrence free survival rate of the HCC patients (Figure 1B, right; the drop at 70 months was due to recurrence of one patient among remaining 3 patients). Furthermore, miR-148a expression was significantly associated with tumor node metastasis (TNM) stage (p<0.001) and α-fetoprotein levels (p<0.001) (Table 1).

**Table:** Table 1. Correlation between miR-148a levels and clinico-pathological characteristics in human primary HCC specimens

miR-148a expression			
	low (n=30)	high (n=29)	p-value
Age Mean ± SD	52.8±10.05	56.1±11.02	0.470^1^
Gender Male Female	24 6	18 11	0.128^2^
Tumor size (cm) ≤5 >5	16 14	19 10	0.341^2^
Tumor stage* TNM I TNM II-III	10 20	24 5	<0.001**^2^
Vascular invasion No Yes	13 17	27 2	<0.001**^3^
Satellite nodule No Yes	27 3	27 2	0.669^3^
E-S grade (MC) I-II III- IV	20 10	24 5	0.156^2^
AFP (ng/ml) ≤20 >20	7 23	18 11	0.003**^2^
Etiology HBV HCV Alcohol Idiopathic	25 1 1 3	22 2 2 3	0.759^3^

1 modified AJCC

2 p<0.01,

^1^Student's t- test,

^2^Chi-square test,

^3^Fisher's exact test

Abbreviation: AFP, α-fetoprotein; AJCC, American Joint Committee on Cancer; HBV, hepatitis B virus;

To elucidate the molecules involved in HCC progression, we attempted to find miR-148a target(s) responsible for the induction of metastatic phenotype. Of 698 targets predicted by TargetScan algorithm, 16 genes of ‘cell migration’ ontology were used for network analysis; UBC, FGF2, and TGFBR1 were extracted from STRING database and were recently published data as the ‘core genes’ having the greatest interaction partner genes (Figure 1C, left). The gene network analysis enabled us to predict USP4 as a molecule that bridges UBC and TGFBR1, and S1P1 as a molecule that links UBC and FGF2. Moreover, highly conserved miR-148a recognition sites were present in the 3'-UTR regions of USP4 and S1P1 mRNAs (Figure 1C, right). Thus, we focused USP4 and S1P1 as the putative targets of miR-148a for HCC progression. In the immunoblottings, USP4 protein levels were up-regulated to a greater extent in HCC samples (Figure 1D, left); 18 out of 59 patients with HCC (31%) exhibited higher levels of USP4 by at least a two-fold as compared with the corresponding NT. S1P1 protein levels were also higher in HCC than NT (Figure 1D, right). Immunohistochemical analyses showed that USP4 and S1P1 levels were also enhanced in the primary HCC as compared to NT (Figure 1E). In the HCC samples, the levels of USP4 inversely correlated with those of miR-148a, whereas those of S1P1 did not (Figure 1F). These results provide evidence that low and high miR-148a expression may discriminate the survival rates of HCC patients, and MVI versus non-MVI, and that of the new targets up-regulated due to miR-148a dysregulation, a significant inverse correlation existed between miR-148a and USP4.

### Overexpression of USP4 or S1P1 in mesenchymal-type liver tumor cells

**Figure 2:**
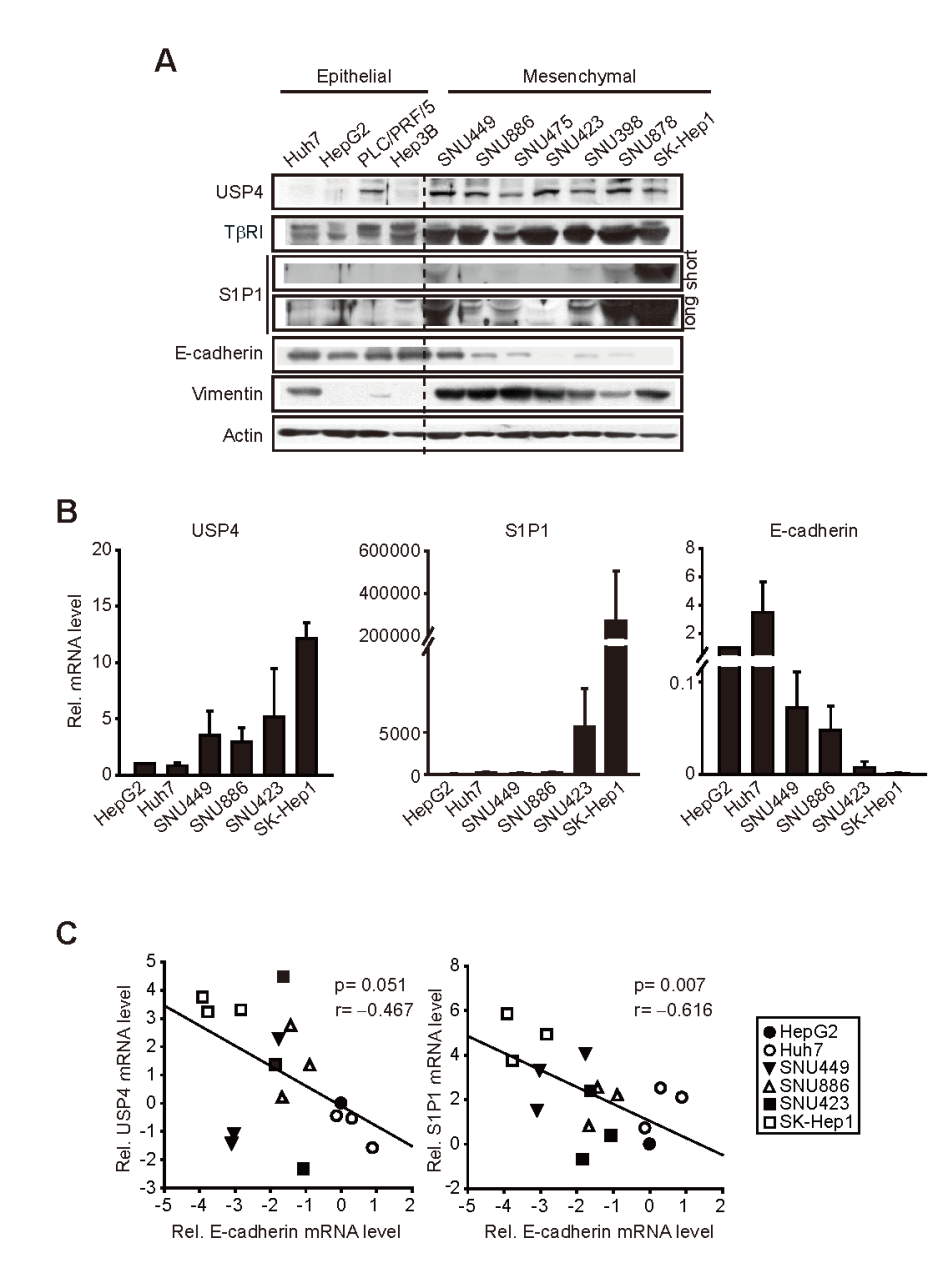
Comparisons of USP4, S1P1 and E-cadherin levels in liver-tumor cell lines (A) Immunoblottings for USP4, S1P1 and E-cadherin. E-cadherin and vimentin were used as the criteria to determine epithelial- or mesenchymal-phenotype. (B) qRT-PCR assays for USP4, S1P1 and E-cadherin transcripts. (C) Correlation analyses. The relative USP4 or S1P1 mRNA levels were plotted against those of E-cadherin (E-cadherin in HepG2=1). Actin was used as control. For B and C, data represented the mean ± S.E. of at least 3 separate experiments.

Mesenchymal-type HCC exhibits greater metastatic and invasive abilities [17]. To associate increases of the identified targets with tumor aggressiveness, immunoblottings were done on epithelial-phenotype or mesenchymal-phenotype liver-tumor cells; HepG2, Huh7, PLC/PRF/5, Hep3B, SNU449, SNU886, SNU475, SNU423, SNU398, SNU878, and SK-Hep1. USP4 levels were much higher in the cells having a mesenchymal-phenotype than those having an epithelial-phenotype (Figure 2A). USP4 promotes TGF-β signals by stabilizing TGF-βtype I rceptor (TβRΙ) [8]. As expected, TβRΙ levels were elevated in the cells as USP4 increased. In addition, S1P1 was overexpressed in the mesenchymal-type cells. Mesenchymal characteristics of the cells were validated by a deficiency in E-cadherin and vimentin overexpression. Consistently, those having a mesenchymal-phenotype exhibited higher expression of USP4 or S1P1 mRNA (Figure 2B). When the relative levels of USP4 or S1P1 mRNA were plotted against E-cadherin mRNA in selected cell lines, inverse correlations were found (Figure 2C). In contrast to S1P1, the expression levels of other isoforms (S1P2-5) were not much different among the cell lines examined (Supplementary Figure S1). All of these results showed that USP4 and S1P1 levels were greater in liver-tumor cells having a mesenchymal-phenotype.

### USP4 and S1P1 as direct targets of miR-148a

**Figure 3:**
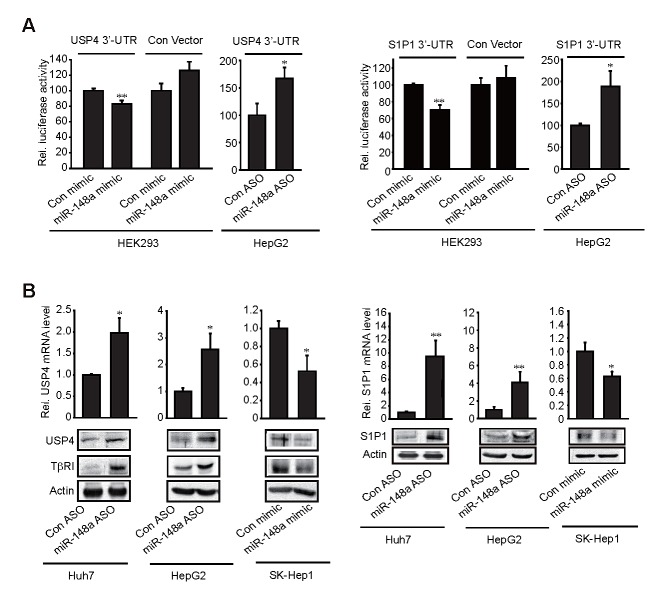
Inhibition of USP4 or S1P1 translation by miR-148a (A) 3'-UTR reporter assays. Indicated cells were transfected with control mimic (or ASO) or miR-148a mimic (or ASO) in combination with reporter construct. (B) The effect of miR-148a ASO or mimic on USP4 and S1P1 expression.Data represent the mean±S.E. of at least 3 separate experiments (significantly different from transfection control, *p<0.05, **p<0.01).

As a continuing effort to verify the targets of miR-148a, we determined whether miR-148a inhibits USP4 and S1P1 by directly interacting with the 3'-UTR of the mRNAs. miR-148a mimic transfection decreased luciferase expression from Luc-USP4-3'-UTR construct in HEK293 cells. In addition, miR-148a ASO transfection increased luciferase activity from the construct in HepG2 cells, which corroborates the inhibitory effect of miR-148a on USP4 (Figure 3A, left). Similar results were obtained using Luc-S1P1-3'-UTR construct (Figure 3A, right). We further assessed the effects of miR-148a modulations on USP4 and S1P1 levels in three different liver-tumor cell lines. As expected, transfection of either Huh7 or HepG2 cells with miR-148a ASO resulted in the induction of USP4, which was accompanied by the stabilization of TβRI (Figure 3B, left). In SK-Hep1 cells, miR-148a mimic transfection attenuated the band intensities of USP4 and TβRI. USP4 mRNA levels were also correspondingly changed by the modulations of miR-148a, suggesting that miR-148a may destabilize USP4 mRNA. miR-148a levels were confirmed after transfection of miR-148a mimic or ASO into liver tumor cells. In addition, we measured the effect of miR-148a ASO or mimic transfection on the levels of S1P1 in the cell lines. miR-148a ASO transfection promoted the induction of S1P1 in Huh7 or HepG2 cells, whereas miR-148a mimic transfection diminished it in SK-Hep1 cells (Figure 3B, right). S1P1 mRNA levels were also similarly changed in the cells. Our results provide evidence that miR-148a has the ability to directly inhibit de novo synthesis of USP4 and S1P1.

### Tumor cell growth and migration after modulation of USP4 or S1P1

**Figure 4:**
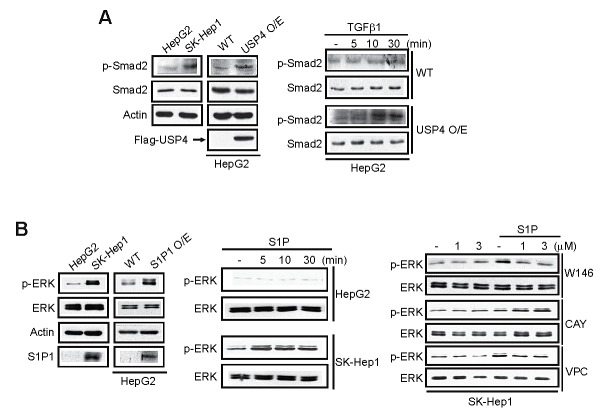
The effects of USP4 or S1P1 overexpression on the phosphorylation of Smad2 or ERK (A) Immunoblottings for phosphorylated or total Smad2. WT cells or those stably overexpressing USP4 were treated with vehicle or TGF-β1 (2.5 ng/ml).(B) Immunoblottings for phosphorylated or total ERK. Immunoblottings were done on indicated cells or HepG2 cells stably overexpressing S1P1 (left). Cells were treated with vehicle or 1 μM S1P (middle). SK-Hep1 cells were treated with S1P receptor antagonist for 1 h and continuously exposed to S1P for 5 min (right). W146, a specific S1P1 antagonist; CAY10444, an S1P3 antagonist; and VPC23019, an antagonist of S1P1 and S1P3. for indicating time point.

Subsequently, we assessed whether signaling pathways activated by ligand activation were fortified in the cell lines or those having enforced expression of USP4 or S1P1. In this experiment, we chose HepG2 and SK-Hep1 as the representative cells. TGF-β receptor activation leads to Smad2 and Smad3 phosphorylation, which depends on TβRI [8]. Since Smad2 phosphorylation is frequently used to assess the activation status of TGF-β signaling, we measured p-Smad2 as an indicator of TβRI activity. Basal p-Smad2 levels were higher in SK-Hep1 than HepG2 (Figure 4A, left). Similarly, HepG2 overexpressing USP4 showed the greater band intensity in p-Smad2 than did wild type HepG2. Moreover, the ability of TGF-β to activate Smad2 was augmented by USP4 overexpression (Figure 4A, right). As a continuing effort to find the effect of S1P1 on tumor cell growth, we determined extracellular signal-regulated kinase (ERK) activation, and found that the basal ERK phosphorylation was higher in SK-Hep1 than HepG2, which paralleled S1P1 levels (Figure 4B, left). In addition, S1P treatment facilitated ERK phosphorylation to a greater extent in SK-Hep1 than HepG2 (Figure 4B, middle). We found that S1P activation of ERK in SK-Hep1 was attenuated by W146, a specific S1P1 antagonist, but not by CAY10444, an S1P3 antagonist. VPC23019, an S1P1 and S1P3 antagonist, similarly inhibited ERK phosphorylation by S1P (Figure 4B, right). These results showed that overexpression of USP4 or S1P1 contributes to activating signaling pathways downstream from cell surface receptors.

**Figure 5:**
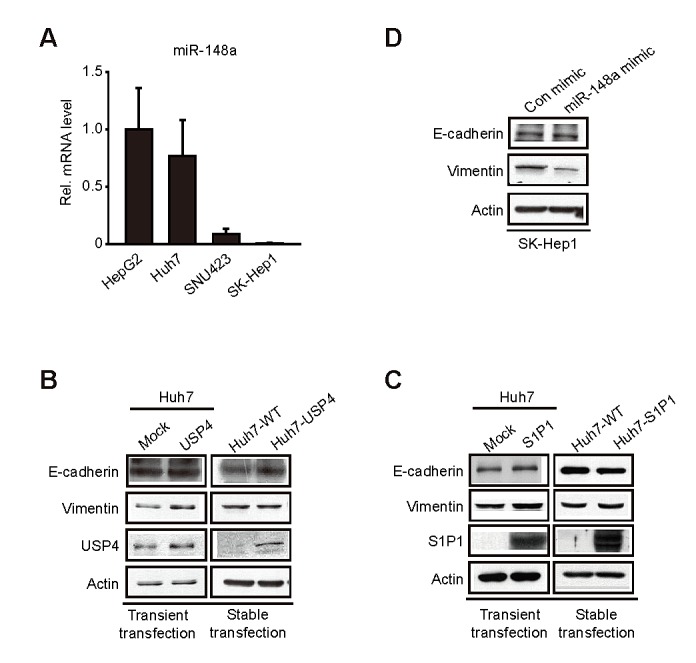
The effect of miR-148a, USP4, and S1P1 on the expression of EMT markers (A) qRT-PCR assays for miR-148a in liver-tumor cells.(B) Immunoblottings for E-cadherin and vimentin in Huh7 cells transfected with the construct encoding for USP4. Huh7 cells were transfected with the construct (1 μg, for 12 h) (left). Stably transfected cells were selected by culturing in the presence of Geneticin (700 μg/ml, for 14 days; the media were changed every 2 days). The selected multiple colonies were used for assays.(C) Immunoblottings for E-cadherin in Huh7 cells similarly transfected with S1P1 as described above.(D) Immunoblottings for E-cadherin and vimentin in SK-Hep1 cells repetitively transfected with miR-148a mimic. The cells were transfected with 100 nM control mimic or miR-148a mimic for 48 h, and this procedure was repeated four more times.

We next assessed whether overexpression of the identified targets by miR-148a dysregulation causes epithelial-mesenchymal transition (EMT) using cell models. As expected, miR-148a levels were lower in SK-Hep1 and SNU423 (mesenchymal-type) than in Huh7 and HepG2 (epithelial-type) (Figure 5A). Neither transient nor stable overexpression of USP4 inhibited E-cadherin in Huh7 cells (Figure 5B). Consistently, cell morphology was unchanged (data not shown). Similar results were obtained using HepG2 cells (data not shown). Enforced expression of S1P1 also resulted in the same outcomes (Figure 5C) although S1P1 knockdown slightly decreased vimentin and Zeb levels (data not shown). Moreover, repetitive transfections with miR-148a minimally changed EMT marker levels (Figure 5D). These results support the notion that either USP4 or S1P1 overexpression may be necessary, but not sufficient, to facilitate EMT.

**Figure 6:**
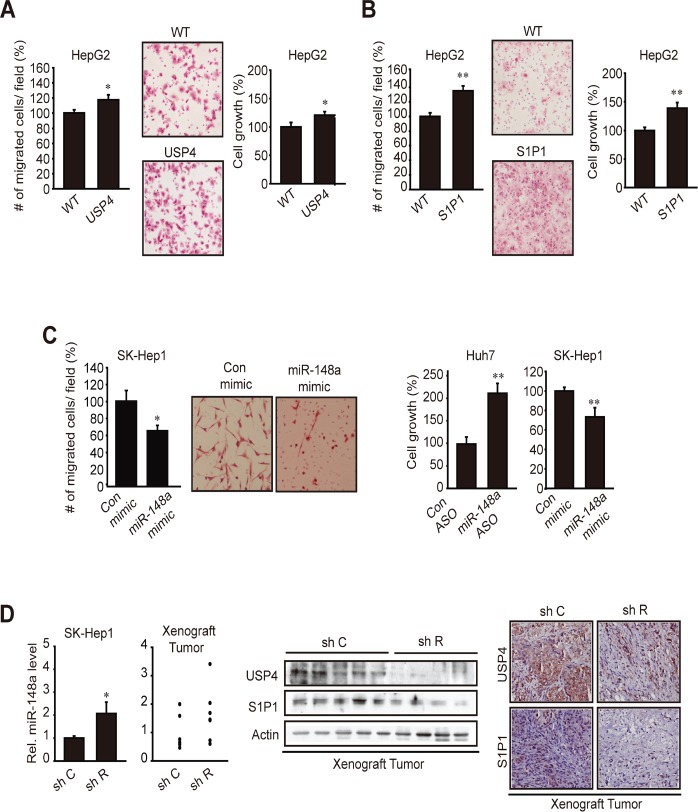
The effect of USP4 or S1P1 overexpression on tumor cell migration and growth (A) The effect of stable overexpression of USP4 on cell migration and growth. Indicated cells were subjected to transwell migration assay or MTT assays for 24 h. (B) The effect of stable overexpression of S1P1 on cell migration or growth.(C) The effects of miR-148a modulations. Cells were transfected with control ASO or miR-148a ASO for 72 h, or with control or miR-148a mimic for 48 h, and were subjected to MTT (24 h) or transwell migration (17 h) assays.(D) Analyses of miR-148a, USP4, and S1P1 in xenograft tumors. qRT-PCR assays were done on SK-Hep1 cells transfected with control shRNA (sh C or shRNA directed against Gα12 (sh R) (N=3). sh C or sh R cells (1×10^7^ each) were injected into the left flank of athymic nude mice. Of 8 or 9 tumors formed in each group at 8 weeks, qRT-PCR assays were done on the samples with sufficient amount (N=6 each). Tumor samples were used for immunoblottings (N=8-9 each) or immunohistochemistry (×200, representative figures were shown; N=4 each). For A-D, data represent the mean±S.E. of 3 separate experiments (significantly different from transfection control or WT control, *p<0.05, **p<0.01).

In the subsequent experiments, we determined the role of USP4, S1P1, or miR-148a in tumor cell migration and growth. Cell-migrating capability was assessed using transwell migration assays. As expected, stable overexpression of USP4 facilitated migration and proliferation of HepG2 cells (Figure 6A). Likewise, stable overexpression of S1P1 had similar effects (Figure 6B). Modulations of miR-148a using mimic or ASO altered the migrating or growing capability of Huh7 and SK-Hep1 cells (Figure 6C). In addition, we verified the inverse relationship between miR-148a and USP4 (or S1P1) in a tumor xenograft model. In this experiment, we took an advantage of the ability of Gα*_12_* to promote liver tumor EMT and of the fact that this effect accompanies decrease of miR-148a (Yang et al, submitted). Gα*_12_* -depletion using a shRNA approach (shR) increased miR-148a level in SK-Hep1 cells (mesenchymal-type), which was lessened but maintained in tumors formed from the cell (Figure 6D). In the xenograft tissues, USP4 or S1P1 levels were notably diminished, showing that an approach modulating the upstream regulator of miR-148a creates the expected changes in USP4 or S1P1 expression *in vivo*. Decreases in the overall tumor growth rate and tumor weight by sh R were confirmed in a separate study (Yang et al, submitted). Our results demonstrate that overexpression of USP4 and S1P1 due to miR-148a dysregulation contributes to the growth advantage or migrating capability of liver tumor.

### miR-148a, USP4, and S1P1 levels in a patient-derived tumorgraft model

**Figure 7:**
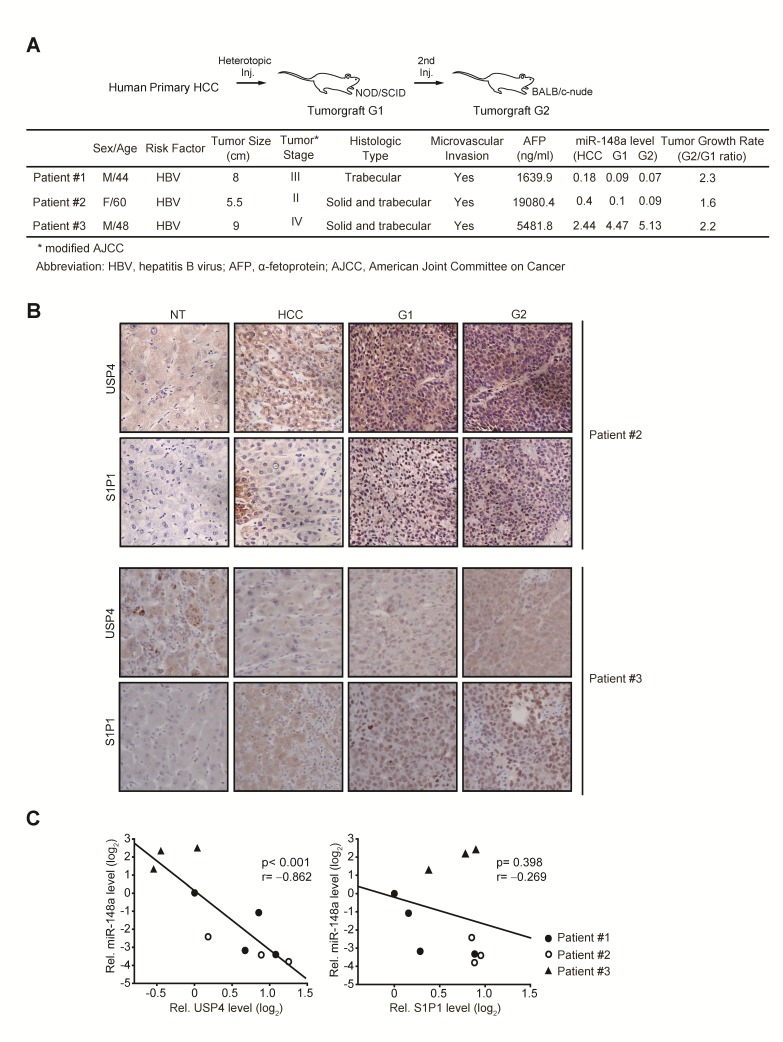
Analyses of miR-148a and its target expression levels in original HCC or engrafted tumors (A) Clinical and pathological characteristics of donor patients. miR-148a levels were assessed using qRT-PCR assays and were normalized relative to U6 small RNA. miR levels in tumors were compared with NT samples. One or two paraffin blocks were made from G1 or G2 tumors, and the rest samples were used for tumor passages.(B) Immunohistochemical analyses for USP4 or S1P1 in original HCC or engrafted tumors. Shown above are the figures obtained from patient #2 and #3 (×200). The results from patient #1 and #2 were comparable to each other.(C) Correlation analyses between miR-148a and USP4 (or S1P1). U6 small RNA was used as control. (NT=1)

To verify biological relevance of the identified targets in a clinical situation of HCC more in depth, we used a heterotopic patient-derived HCC xenograft animal model (Figure 7A). In the two primary HCC samples out of three, miR-148a levels were markedly diminished as compared to respective NT liver tissues. Histopathological examinations confirmed the characteristics of original tumors after each passage of tumorgraft (data not shown, JW Park). Immunohistochemical analyses revealed that USP4 and S1P1 levels were both enhanced with miR-148a decrease in two HCC samples (#1 and #2) as compared to NTs and were further increased in their G1 or G2 tumors of xenograft (Figure 7B). In patient #3, miR-148a levels were rather increased in the HCC compared to NT and further enhanced in the G1 or G2 tumor. Consistently, USP4 expression was not increased in the HCC or G1/G2 tumors. S1P1 expression tended to increase. In the combined analyses, the levels of USP4, but not S1P1, was inversely correlated with miR-148a expression (Figure 7C). Collectively, USP4 is overexpressed as a result of miR-148a dysregulation, which may reflect a shift in the expansion phase of tumorgraft.

## DISCUSSION

Our findings showed for the first time that the overall survival, and the recurrence free survival rates of HCC patients were discerned by the level of miR-148a. Moreover, miR-148a dysregulation may discriminate MVI versus non-MVI and tumor size. Hence, dysregulation of miR-148a may contribute to progression of HCC to advanced stages. In this study, we identified overexpression of USP4 or S1P1 in the human HCC samples as a consequence of miR-148a dysregulation. Computational algorithms and network analyses also indicated USP4 and S1P1 as the ‘core genes’ having the greatest interaction partners for migration ability. The ability of miR-148a to directly inhibit de novo synthesis of USP4 or S1P1 was supported by the results of cell-based assays. However, decreases of miR-148a in human specimens correlated with the changes in USP4, but not S1P1. Therefore, USP4 was the bona fide target of miR-148a. miR-148a is also down-regulated in other tumors such as colorectal and gastric cancers [18,19]. In addition, miR-148a levels in endometrial cancer were lower than in matched normal tissue fibroblasts [20]. The findings that miR-148a transfection attenuated CD90 and CD44 (cancer stem cell markers) expression in HCC [21] along with the fact that miR-148a as a hepatospecific miRNA is highly expressed in adult liver [22] suggest that the decrease of miR-148a in HCC is likely to reflect repression of the miRNA in cancer cells rather than stromal cells.

USPs belong to the largest deubiquitinase family and may affect post-translational modifications of proteins [7]. Altered expression of USP may affect TGF-βactivity through ubiquitin modification of receptor or signaling molecules [23]. In particular, USP4 pre-activates and/or persistently increases TβRI stabilization. Our findings that miR-148a dysregulation correlated with USP4 in the tumor samples and that patients having high USP4 showed a tendency to have high TNM stage (TNM stage II-III) support the role of USP4 in the transition of HCC to a malignant phenotype. Moreover, USP4 was up-regulated in mesenchymal-phenotype liver-tumor cells. In addition, TβRI expression is controlled by miR-140-5p [24]. Twenty miRNAs including miR-148a had been shown to be down-regulated in HCC with distant metastasis [25]. Thus, USP4 overexpression elicited by dysregulation of miR-148a and other miRNA(s) may facilitate HCC progression.

TGF-β induced signals exert pro-invasive and pro-metastatic responses possibly through EMT [26]. TβRI stabilized downstream from TGF-β signaling causes the phosphorylation of Smad2 and 3, the transcription factors responsible for target gene induction [8], as verified by our result that USP4 fortified Smad2 phosphorylation and promoted migration and proliferation of hepatoma cell. EMT, an event characterized by a loss of E-cadherin, contributes to metastatic property of cancer cells. Our findings indicated that USP4 and S1P1 were up-regulated and inversely correlated with E-cadherin in mesenchymal-typed cells. Consistently, miR-148a levels were down-regulated in the cells, being consistent with a recent report [27]. However, our findings showed that USP4 or S1P1 overexpression was insufficient to facilitate EMT, implying that a set of dysregulated miRNAs (e.g., miR-125b and miR-101) may contribute to phenotype changes, depending on the stage and progression of HCC [28,29].

S1P, an inflammatory lipid modulator, stimulates cancer cell survival, growth, migration, and neovascularization through S1P1-5 receptors [10,30]. In several tumors, S1P and its receptor levels are up-regulated (e.g., S1P1 and/or S1P3 in breast and ovarian cancers) [12,31]. Nevertheless, no information had been available on the expression of S1P receptors in HCC. Here, we showed that S1P1 expression was up-regulated in HCC. More importantly, S1P1 levels were most significantly elevated in liver tumor cells having a mesenchymal-phenotype, implying its role in cancer aggressiveness. Although levels of most other S1P receptor isoforms were higher in SK-Hep1 than HepG2, increase of S1P1 was the most substantial. Hence, it is expected that S1P1 induction in concert with sphingosine kinase 1 and S1P production in tumor microenvironments may contribute to the progression of HCC to more aggressive phenotype [32].

MEK/ERK pathway is involved in tumor cell proliferation and migration, promoting metastatic processes [33]. Here, we found that ERK was activated to a greater extent in a mesenchymal-type cell. Consistently, S1P1 overexpression enhanced ERK activation in an epithelial-type cell, being consistent with the finding that ERK activities were increased in breast cancer overexpressing S1P1 [12]. Consistently, S1P1 induction by miR-148a dysregulation promoted proliferation or migration of hepatoma cells, and the causal effect relationship was strengthened by the experiments using siRNA (data not shown) and miR-148a mimic. Overall, it is likely that ligand activation of S1P1 boosts the signals from tyrosine kinase receptors through cross-talks [34,35]. However, S1P1 levels in the HCC samples were not significantly correlated with miR-148a dysregulation, which may be due to differences in the miRNA affinity to different targets and the nature of multiple miRNAs interaction with a single target. Also, S1P1 overexpression would be more closely associated with metastasized tumor cells since S1P receptors contribute to chemoattraction [36]. This possibility may have been reflected by the lack of statistical change between miR-148a and S1P1 in our HCC samples obtained from the patients who had no distant metastasis but only MVI in a certain fraction.

Patients whose tumors showed high engraftment rates had poor overall survival and high metastatic potential [37]. Our result shown in a heterotropic patient-derived HCC xenograft model verified the overexpression of USP4 in G1 and G2 tumors when miR-148a was dysregulated. In this model, S1P1 levels were not significantly correlated with decrease of miR-148a. Hence, it is highly likely that USP4 induction is closely linked to miR-148a dysregulation for a shift in the expansion phase of tumorgraft. Collectively, our results support the concept that dysregulation of miR-148a is associated with the poor prognosis of HCC and may account for the tumor progression to advanced stages, and that, of the newly identified targets, USP4 overexpression may contribute to HCC progression towards more aggressive feature presumably by facilitating TGF-β signaling pathways, growth advantage and migrating capability.

A miRNA can regulate many targets. Other known targets of miR-148a include bcl-2, rho-associated protein kinase 1, c-Myc, and HPIP in cancers including gastric cancer, colorectal cancer, and HCC [6,38-40]. So, USP4 and S1P1 may work with the aforementioned molecules for the favor of tumor growth and metastasis. Our findings may provide key information on the role of miR-148a dysregulation and the relevant changes in USP4 for HCC progression and/or malignancy.

## METHODS

### Materials

Anti-USP4, anti-S1P1 (EDG1), and anti-S1P3 (EDG3) antibodies were purchased from Santa Cruz Biotechnology (Santa Cruz, CA). Anti-USP4 and anti-S1P1 antibodies used for immunohistochemistry were provided from Abcam (Cambridge, UK). Anti-β-actin antibody was supplied from Sigma-Aldrich (St. Louis, MO). Antibodies directed against ERK, Smad2, p-ERK, and p-Smad2 were obtained from Cell Signaling Technology (Beverly, MA). Anti-E-cadherin antibody was supplied from BD Biosciences (San Jose, CA), whereas horseradish peroxidase-conjugated goat anti-rabbit and goat anti-mouse IgGs were from Zymed Laboratories (San Francisco, CA). W146 and VPC23019 were purchased from Avanti Polar Lipids (Alabaster, AL), whereas SEW2871 and CAY-10444 were from Cayman Chemical (Ann Arbor, MI).

### Applications of HCC patient samples

Fifty nine paired samples of HCC tumor and surrounding NTs collected during the period between 2006 and 2009 were supplied from the Asan Medical Center (Seoul, Korea) after review and approval by institutional review board (#2012-0133). Informed consents from the patients were obtained before operations. Nineteen tumor samples were classified as a MVI group, whereas forty were as a non-MVI group. Tumors were classified according to the World Health Organization pathologic classification system. After resection, fresh surgical specimens were immediately snap-frozen in liquid nitrogen and stored at −80°C.

For a tumorgraft animal model, three paired HCC samples and surrounding NTs approved by institutional review board of National Cancer Center, Korea (#NCCNCS-12-593) and Seoul National University (E1401/001-003) were used. Surgical specimens of HCC freshly obtained from patients were washed with RPMI 1640 medium and minced under sterile conditions. Approximately 200 μl of individual HCC tumors mixed with matrigel were subcutaneously implanted in NOD/SCID mice (1st passage, G1). Tumor growth was monitored, and dimensions of xenografts were measured twice a week. Tumor specimens that grew to a size of 1 cm3 were retrieved and the samples were again engrafted into recipient BALB/c-nude mice (2nd passage, G2). Successful engraftments were confirmed by tumor growth measurements and histopathological examinations using hematoxylin and eosin staining.

### Integrative network analysis

Gene targets of miRNA-148a with conserved seed-match were predicted by TargetScan algorithm. Gene ontology clustering analysis was performed using DAVID 6.7 software [41,42]. Gene interaction analysis between the clustered genes was achieved according to STRING v9.1 database [43], and visualization was done using Cytoscape 3.0.0 software [44].

### Immunohistochemistry

Tumor specimens were fixed in 10% formalin, embedded in paraffin, cut into 4 μm thick sections, and mounted on slides. Tissue sections were immunostained with antibodies directed against USP4 or S1P1. Briefly, the sections were deparaffinized and were incubated with anti-USP4 antibody (1:100) or anti-S1P1 antibody (1:100) for 2 h, followed by polink-2 plus polymer HRP detection (GBI, WA). Micrographs obtained from immunohistochemistry were analyzed by ImageJ software.

### Cell culture

Huh7, PLC/PRF/5, Hep3B, SK-Hep1, SNU449, SNU886, SNU475, SNU423, SNU398, and SNU878 cell (human HCC) cells were cultured in Dulbecco's modified Eagle's medium (DMEM) (Gibco, Gaithersburg, MD). HepG2 and HEK293 cells were cultured in HyClone DMEM Media (Logan, UT), whereas Huh7 and PLC/PRF/5 were done in RPMI-1640 (Gibco, Gaithersburg, MD). All media contained 10% fetal bovine serum (FBS), and 5% penicillin–streptomycin at 37°C in a humidified atmosphere containing 5% CO2. For all experiments, the cells were grown to 80-90% confluence and were deprived of serum for 15 h.

### Immunoblot analysis

Proteins were separated by 7.5% SDS-polyacrylamide gel electrophoresis and transferred onto nitrocellulose membrane (Millipore, Bedford, MA). The membrane was blocked with 5% non-fat dried milk in TBST (20 mM Tris-HCl, 150 mM NaCl, and 0.1% Tween 20, pH 7.5) for 1 h and incubated overnight with each primary antibody at 4°C. After washing with TBST buffer, membranes were incubated with secondary antibodies for 1 h at room temperature. The protein bands were visualized using an ECL chemiluminescence system (Amersham, Buckinghamshire, UK). Equal loading of samples was verified by immunoblotting for β-actin. Band intensities were quantified using Adobe Photoshop CS5 (Adobe Systems, San Jose, CA).

### Reverse transcription- and quantitative RT-PCR assays

Total RNA was reverse-transcribed. The resulting complementary DNA was amplified by polymerase chain reaction (PCR) using the PCR Master kit (Roche, Mannheim, Germany). Quantitative real-time (qRT) PCR was performed using ABI StepOne plus Real-Time PCR System and 48-well optical reaction plates (Applied Biosystems, Foster City, CA). The following primer sequences were used: human GAPDH, 5'-GAAGATGGTGATGGGATTTC-3' and 5'-GAAGGTGAAGGTCGGAGTC-3'; human actin, 5'-CTCTTCCAGCCTTCCTTCCTG-3' and 5'-CAGCACTGTGTTGGCGTACAG-3'; human S1P1, 5'-ATTACTTTAACTGGTAGGGAACG-3' and 5'-AAGACATCTCTCGGTTTAATTGC-3'; human S1P2, 5'-GGCCTTCGTAGCCAATACCT-3' and 5'-TGCCATACAGCTTGACCTTG-3'; human S1P3, 5'-GCCACCATTTCCACTAGGAG-3' and 5'-GCATATTGGTGCACATTGGT-3'; human S1P4, 5'-GAGAGCACCCTGGTGTGG-3' and 5'-CATGATCGAACTTCAATGTTGC-3'; human S1P5, 5'-CCACGACTGTCTTCCCAAGT-3' and 5'-CAAGCAGAACGTCAATTCCA-3'; human E-cadherin, 5'-TGAAGGTGACAGAGCCTCTGGA-3' and 5'-TGGGTGAATTCGGGCTTGTT-3'; and human USP4, 5'-ACCATTGCAACCATCGAGAA-3' and 5'-TTTTGACTGCAAGGTCTGCC-3'. For qRT-PCR assays of miRNA, complementary DNAs were generated from equal amounts of total RNA per sample (1 μg) using the miScript Reverse Transcription kit (Qiagen GmbH, Hilden, Germany). The reaction mixture containing reverse transcription product, 2×QuantiTect SYBR Green PCR Master Mix, 10×miScript Universal Primer, and primer was incubated at 95°C for 15 min, followed by 40 amplification cycles of 94°C for 10 s, 55°C for 30 s, and 70°C for 30 s. The threshold cycle (Ct) was defined as the fractional cycle number at which the fluorescence passed the fixed threshold. Transcripts of U6 small RNA were also quantified using the Hs_RNU6B_2 miScript Primer Assay (Qiagen, Hilden, Germany) for normalization of miRNA levels. The sequence of human miR-148a is 5'-UCAGUGCAUCACAGAACUUUGU-3'.

### Transfection of miRNA mimic or antisense oligonucleotide

Synthetic miRNA duplexes were synthesized, as previously described [42]. The following sequences were used: miR-148a mimic, 5'-UCAGUGCACUACAGAACUUUGU-3' (guide) and 5'-AAAGUUCUGUAGUGCACUGACU-3' (passenger). 2'-O-methyl control ASO and miR-148a used for cell culture experiments were custom-synthesized from Bioneer (Daejeon, Korea). The following primer sequences were used: 2'-O-methyl control ASO, 5'-CCTTCCCTGAAGGTTCCTCCTT-3'; and 2'-O-methyl miR-148a, 5'-ACAAAGUUCUGUAGUGCACUGA-3'. The cells in each well (6-well plates) were transiently transfected with 100 pmoles of control mimic (Santa Cruz, CA) or miR-148a mimic, or 100 pmoles of 2'-O-methyl miR-148a ASO or respective negative control ASO using FuGENE HD Reagent (Roche, Indianapolis, IN).

### USP4 or S1P1 3'UTR reporter assays

The plasmids contain firefly luciferase fused to the 3'UTR of human USP4 or S1P1, and Renilla luciferase functioning as a tracking gene: Luc-USP4-3'-UTR and Luc-S1P1-3'-UTR (Product ID: HmiT055448-MT01 and HmiT00454-MT01, GeneCopoeia, Rockville, MD). Briefly, HEK293 cells were transfected with miR-148a mimic and USP4 (or S1P1) 3'UTR reporter construct in an Opti-MEM medium in 6-well plates, and the medium was changed with DMEM-high glucose 12 h after transfection. The cells were incubated for 36 h before harvest. Similarly, HepG2 cells were transfected with 2'-O-methyl miR-148a and the 3'UTR reporter, and were incubated for 24 h. The cells were harvested 48 h after change of medium. Firefly and Renilla luciferase activities were measured sequentially using a dual luciferase assay kit (GeneCopoeia, Rockville, MD). Activities were normalized to those of Renilla luciferase and expressed as relative luciferase activity units.

### Establishment of stable cell lines

Either Myc-DDK-tagged-USP4 (RC224586, Origene, Rockville, MD) or S1P1 (human EDG1, EDG0100000, Missouri S&T cDNA Resource Center) expression vector was transfected into HepG2 or Huh7 cells using FuGENE HD Reagent (Roche, Indianapolis, IN), and stably transfected cells were selected using G418 (700 μg/ml) (GIBCO, Carlsbad, CA). PCMV or PCDNA3.1 was used as control vector.

### MTT assays

Indicated cells transfected with control ASO or miR-148a ASO for 72 h (or with control mimic or miR-148a mimic for 48 h), wild type HepG2 or those overexpressing USP4 (or S1P1) were plated at a density of 1×104 cells per well in a 48-well plate. Viable cells were stained with 0.25 mg/ml 3-(4,5-dimethylthiazol-2-yl)-2,5-diphenyl-tetrazolium bromide (MTT) for 1 h. The media were then removed, and formazan crystals produced were dissolved by the addition of dimethylsulfoxide (absorbance, 540 nm).

### Transwell migration assays

Cell migration assays were done using a 24-well transwell unit with polycarbonate membrane (8 μm pores; Costar, Corning, Inc., Corning, NY); 5×10^4^ cells in a medium without serum were added into the upper chamber of the insert, and the lower chamber contained a medium containing 5% fetal bovine serum as a chemoattractant. After incubation for 17 h, the insert was fixed with methanol and was stained with Hematoxylin and Eosin. The cells were counted with a microscope.

### Statistical analysis

Data were shown as the mean±S.E. from at least three separate experiments. To assess significant differences between two groups, Student's t-test was performed. Coefficients of correlation (r) were determined by the Pearson correlation method. The Kaplan–Meier method was used for survival analysis. Chi-square tests were used to compare the associations of categorical variables with low or high miR-148a expression. Fisher's exact test was used for categorical variables with small expected numbers. For age, p-value was determined using Student's t-test. Statistical calculations of Pearson's correlation and Kaplan–Meier method were performed using SPSS 20.0. P values < 0.05 were considered statistically significant.

## SUPPLEMENTARY FIGURE


