# Supplementary material for: microRNA-148a dysregulation discriminates poor prognosis of hepatocellular carcinoma in association with USP4 overexpression
Source: Oncotarget. 2014 Apr 25;5(9):2792–806. doi: 10.18632/oncotarget.1920 (PMC4058045; doi:10.18632/oncotarget.1920)
Supplement: Supplementary file 1 [file oncotarget-05-2792-s001.pdf]

microRNA-148a dysregulation discriminates poor prognosis of hepatocellular carcinoma in association with USP4 overexpression

Supplementary Material

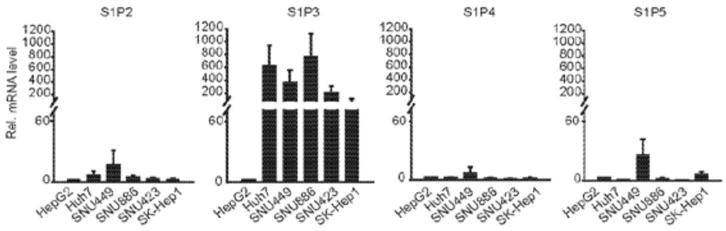

Supplementary Figure S1. qRT-PCR assays for S1P2-S1P5 mRNAs. Data represent the mean S.E. of 3 separate experiments (mRNA level in HepG2 = 1).
